# Selective Colorimetric Determination of Phenylephrine Using a Prussian Blue Nanoparticle-Modified Paper-Based Sensor

**DOI:** 10.3390/bios16060339

**Published:** 2026-06-16

**Authors:** Nihal Ermiş, Nigar Aksöz, Mustafa Oğuzhan Sert

**Affiliations:** Department of Biomedical Engineering, Faculty of Engineering and Natural Sciences, Samsun University, 55420 Samsun, Türkiye

**Keywords:** phenylephrine, Prussian blue nanoparticles, colorimetric sensor, paper-based sensor, digital image analysis

## Abstract

Phenylephrine is a widely used α_1_-adrenergic agonist employed as a decongestant and vasoconstrictor in numerous pharmaceutical formulations. Considering its widespread use and its relevance in biological monitoring and anti-doping control, the development of rapid, sensitive, and reliable analytical methods for its determination has attracted significant attention. A paper-based colorimetric sensor based on Prussian blue nanoparticles was developed for the determination of phenylephrine. Prussian blue nanoparticles were synthesized by the precipitation method, and their structural, morphological, and surface properties were systematically characterized using complementary analytical techniques. The sensing mechanism is based on the reduction in Prussian blue to its colorless form in the presence of phenylephrine, resulting in a decrease in absorbance intensity. Under optimized conditions (pH 6.5 and 5 min incubation time), the colorimetric sensor exhibited a linear response toward phenylephrine over the concentration range of 5–150 µg mL^−1^, with a limit of detection of 1.56 µg mL^−1^ (R^2^ = 0.9986). The sensing system was further integrated into a paper-based platform, enabling visual detection of phenylephrine. Digital image analysis using ImageJ showed a linear response over 5–150 µg mL^−1^ (R^2^ = 0.9884) and a detection limit of 5.37 µg mL^−1^. The sensor’s practical applicability was validated using artificial urine samples, yielding recovery values of 95.87–97.5% and relative standard deviations of 1.15–2.13%. Unlike conventional methods requiring multi-step reactions, this study introduces, for the first time, a simple paper-based colorimetric sensor for phenylephrine detection based on the direct Prussian blue–Prussian white redox transition integrated with digital image analysis.

## 1. Introduction

Colorimetric sensors are analytical systems that detect analytes based on optical signals associated with color changes, offering advantages such as simplicity, low cost, and rapid response. These color variations, arising from interactions between target analytes and sensing components, can be quantitatively measured using UV–Visible (UV–Vis) spectrophotometry and qualitatively observed by the naked eye. Owing to their operational simplicity and suitability for on-site analysis, colorimetric sensors have become widely used in biosensing applications [1,2,3].

The integration of colorimetric sensors into paper-based platforms further enhances their practicality by enabling portable, low-cost, and user-friendly analytical devices. The porous structure of paper allows passive fluid transport through capillary action without the need for external equipment, while hydrophilic channels and hydrophobic barriers enable controlled fluid flow toward defined detection zones [4]. Nanoparticles or reactive compounds immobilized on the paper surface produce easily observable color changes upon interaction with the analyte. This feature increases the importance of paper-based colorimetric sensors in rapid test kits, environmental monitoring systems, and clinical point-of-care (POC) applications. However, several limitations, including analytical accuracy, signal stability, and uncontrolled analyte diffusion across the paper substrate, remain important challenges [5,6].

Prussian blue (PB), a mixed-valence coordination polymer with the chemical formula Fe_4_[Fe(CN)_6_]_3_, is widely recognized for its intense blue color originating from intervalence charge transfer between Fe^2+^ and Fe^3+^ ions. This property, together with its well-defined redox behavior, makes PB an attractive signal-generating component in colorimetric sensing applications. Structurally, PB possesses a face-centered cubic lattice composed of Fe^2+^–CN–Fe^3+^ bridges, and this mixed-valence framework confers the material with distinctive magnetic and electrical properties, as well as redox behavior. The PB exhibits porous structures capable of adsorbing ions and small molecules; consequently, PB has been widely applied in areas such as gas storage, environmental remediation, drug delivery, and sensor design. In addition, PB can be readily synthesized via a simple precipitation method, making it a cost-effective and accessible material [7]. Transforming PB into its nanoparticle form (PB-NPs) significantly increases the surface area of the material, thereby enhancing its reactivity and ion-transport kinetics while improving the stability and reproducibility of its redox behavior. PB-NPs may aggregate upon interaction with analytes, leading to visually detectable color changes on the sensor surface and facilitating colorimetric detection. Furthermore, the surfaces of PB-NPs can be chemically modified, allowing improved selectivity and enabling the detection of multiple analytes [8]. For these reasons, PB-NPs have emerged as highly effective materials for sensor designs that require high sensitivity and strong optical responses.

Recently, Prussian blue (PB)-based colorimetric sensing systems have attracted increasing attention for detecting reducing agents and redox-active analytes due to the reversible Prussian blue–Prussian white (PB–PW) transition. Several studies have utilized this redox behavior for the detection of compounds such as hydrazine, urea, and antibiotics. For example, Nghia et al. developed a portable PB nanoparticle-based colorimetric sensor for hydrazine detection based on the reduction in PB to PW, demonstrating the feasibility of PB-mediated optical sensing of reducing substances [9]. Similarly, Zhang et al. reported a smartphone-assisted, paper-based sensor that exploits the pH-sensitive optical behavior of PB for urea determination [10], while Hu et al. employed PB nanoparticles in dual-mode immunochromatographic assays for antibiotic detection [11]. Despite these advances, previously reported PB-based sensing platforms have mainly focused on environmental analytes, enzymatic systems, or indirect detection mechanisms. To the best of our knowledge, no study has yet reported a paper-based colorimetric sensor specifically designed for phenylephrine detection via the direct PB–PW redox transition, integrated with smartphone-assisted digital image analysis. Therefore, the present study extends the scope of application of PB-based colorimetric sensing to phenylephrine detection using a simple reagent-free optical sensing strategy.

Phenylephrine (PE) is a selective α_1_-adrenergic agonist with sympathomimetic properties. It is commonly used as an ophthalmic solution to induce pupil dilation (mydriasis) during retinal examinations. In cases of low blood pressure (hypotension), particularly those associated with septic shock, phenylephrine is also administered intravenously as a vasopressor to increase blood pressure. In addition, it belongs to the class of nasal decongestants and is widely used to relieve nasal congestion caused by conditions such as the common cold, allergies, and hay fever. Due to its stimulant effects, including the ability to improve nasal airflow and increase alertness by reducing sinus congestion and pressure, phenylephrine may provide an unfair advantage during athletic competition and has therefore been included in the World Anti-Doping Agency (WADA) ProhibitΔd List. Furthermore, the presence of functional groups such as amine and hydroxyl groups in the phenylephrine structure facilitates its interaction with redox-active materials, enabling measurable optical signal changes in colorimetric sensing systems [12,13,14].

Despite the well-established use of Prussian blue nanoparticles in colorimetric sensing, their direct application for phenylephrine detection has not been sufficiently explored, and most existing methods still rely on multi-step procedures or additional reagents. In this study, a novel paper-based colorimetric sensor platform was developed based on the interaction between PB-NPs and phenylephrine. Prussian blue nanoparticles were synthesized via precipitation and systematically characterized prior to integration into the sensing platform. The operational parameters of the colorimetric system were optimized to achieve reliable analytical performance, after which the PB-NPs were immobilized onto a paper substrate to construct the sensing device. The analytical performance of the developed sensor was evaluated for selectivity, sensitivity, linearity, and practical applicability, and its feasibility was further demonstrated through sample analysis. Overall, this work introduces an original sensor design in which PB-NPs function as the colorimetric recognition and signal-generating element, providing a practical, selective, and low-cost strategy for the detection of phenylephrine.

## 2. Materials and Methods

### 2.1. Reagents and Instruments

NaOH (ISOLAB, Eschau, Germany), NaH_2_PO_4_ (Carlo Erba, Milan, Italy), and citric acid (Kimyalab, Ankara, Türkiye) used in this study were obtained from the respective suppliers. All other chemicals were purchased from Sigma-Aldrich (St. Louis, MO, USA) and used without any further purification. Deionized (DI) water (18.2 MΩ·cm) was produced using a Millipore purification system. All solutions were freshly prepared prior to use.

The optical properties of PB-NPs and their interaction with phenylephrine were investigated using a UV–Visible spectrophotometer (Thermomac UVS 502, Chennai, India) over the wavelength range of 400–900 nm. The morphological structure and surface distribution of the nanoparticles were examined by scanning electron microscopy (SEM) (COXEM EM-30 Plus, Daejeon, Republic of Korea), while their crystalline structure was confirmed using an X-ray diffractometer (XRD) (Thermo Scientific ARL X’TRA, Waltham, MA, USA). The elemental composition of PB-NPs was determined by X-ray photoelectron spectroscopy (XPS) (Thermo Scientific K-Alpha, Waltham, MA, USA), and their chemical bonding structure and surface functional groups were analyzed using Fourier transform infrared spectroscopy (FTIR) (Thermo Scientific Nicolet iS10, Waltham, MA, USA). Hydrodynamic diameter, size distribution, and colloidal stability were measured by dynamic light scattering (DLS) (Malvern Nano ZS, Malvern, UK), while surface charge analyses were carried out using a zeta potential analyzer (Malvern Nano ZS, Malvern, UK). To visualize the paper-based sensors, images were captured with a smartphone camera (Samsung S20, 12 MP, Seoul, Republic of Korea). The digital images obtained were analyzed using ImageJ software (version 1.54, National Institutes of Health, Bethesda, MD, USA).

### 2.2. Synthesis of Prussian Blue Nanoparticles (PB-NPs)

The synthesis procedure of the PB-NPs was carried out using an aqueous co-precipitation method. During synthesis, a citrate medium was used to control the reaction pH, stabilize nanoparticle formation, and ensure controlled nanoparticle growth [15,16]. For this purpose, 1 mM FeCl_3_ and 1 mM K_4_[Fe(CN)_6_] solutions were separately prepared by dissolving the respective salts in a 1 mM citric acid solution. Subsequently, the prepared 1 mM K_4_[Fe(CN)_6_] solution was slowly added dropwise to the 1 mM FeCl_3_ solution under continuous stirring at fixed reagent ratios. The reaction mixture was maintained in a closed container at 70 °C for 45 min. During the reaction, the solution color gradually changed from yellow to green, then to a characteristic dark blue, confirming the formation of PB-NPs. After synthesis, the obtained PB-NP suspension was allowed to cool to room temperature, and the nanoparticles were washed by centrifugation at 7800 rpm for 15 min with deionized water. This washing procedure was repeated three times to remove residual reactants and to improve the colloidal purity of the nanoparticles. After purification, the PB-NPs were redispersed in deionized water, stored for subsequent characterization, and later integrated into the paper-based sensor platform developed in this study.

### 2.3. Characterization of PB-NPs

The morphological, structural, chemical, and optical properties of the synthesized PB-NPs were characterized using various analytical techniques to evaluate their suitability for sensor applications and to support their interaction with phenylephrine. The optical behavior of PB-NPs and their response in the presence of phenylephrine were examined by UV–Vis spectroscopy in the wavelength range of 400–900 nm. The morphology and size distribution of the nanoparticles were analyzed using scanning electron microscopy (SEM), while their crystalline structure was confirmed by X-ray diffraction (XRD) using Cu Kα radiation (λ = 1.5406 Å) within a scanning range of 5–50° (2θ). The chemical bonding structure and interaction with phenylephrine were investigated by Fourier transform infrared spectroscopy (FTIR) over 4000–400 cm^−1^, and the surface elemental composition was determined by X-ray photoelectron spectroscopy (XPS). In addition, the hydrodynamic diameter, size distribution, and colloidal stability of the nanoparticles were evaluated by dynamic light scattering (DLS) and zeta potential measurements.

### 2.4. Optimization of the PB-NP-Based Colorimetric Sensor

The optimization of the colorimetric sensor was carried out through systematic investigations to improve the analytical performance of the PB-NP-based sensing system developed for phenylephrine detection. First, the optimal PB-NP volume for analytical measurements was determined by testing different volumes of the synthesized PB-NP colloid and comparing their absorbance responses in the UV–Vis region. An appropriate PB-NP volume was selected that provided a sufficiently intense analytical signal while keeping the absorbance values within the measurable absorbance range. The effect of incubation time on the sensor response was then evaluated by incubating PB-NP and PE solutions at fixed concentrations for 0–30 min, and the absorbance values recorded at each interval were used to determine the time at which the optical response reached a stable state. Subsequently, the effect of pH on sensor performance was investigated by preparing PB-NP and PE solutions at identical concentrations in media with pH values ranging from 3 to 8. The UV–Vis spectra of PB-NP solutions containing phenylephrine were then recorded to evaluate absorbance changes and assess nanoparticle stability and analyte interaction under different pH conditions. Finally, the effect of temperature on the sensor response was investigated by preparing PB-NP solutions containing phenylephrine and analyzing them at 4 °C, 25 °C, 37 °C, and 60 °C. The solutions were prepared at the previously determined optimal pH and equilibrated to the desired temperature. Absorbance values were recorded before and after incubation for the predetermined incubation time. The resulting absorbance differences were used to evaluate the effect of temperature and to identify the optimal operating conditions of the colorimetric sensor.

### 2.5. Determination of the Analytical Performance of the Colorimetric Sensor

The analytical performance of the developed colorimetric sensor for phenylephrine determination was evaluated through calibration analysis, sensitivity and selectivity tests, and the calculation of the limit of detection (LOD) and limit of quantification (LOQ). Phenylephrine-containing PB-NP solutions at different concentrations were prepared under optimized conditions, and absorbance measurements were recorded using a UV–Vis spectrophotometer in the wavelength range of 400–900 nm, with evaluations performed at the characteristic maximum absorbance wavelength of the sensor. For the calibration study, phenylephrine solutions in the concentration range of 0.5–150 µg mL^−1^ were prepared and the corresponding sensor responses were recorded. The relationship between absorbance values and phenylephrine concentrations was analyzed by linear regression to determine the linear range and correlation coefficient. The LOD and LOQ values were calculated using statistical methods based on 10 replicate measurements of the blank solution, where the standard deviation of the blank signal (σ) and the slope of the calibration curve (S) were used according to the equations LOD = 3σ/S and LOQ = 10σ/S [17].

The selectivity of the sensor was evaluated by comparing its response to PE with those of several structurally related and potentially interfering compounds commonly present in biological matrices, including dopamine, ascorbic acid, glucose, and uric acid, each at a fixed concentration of 100 µg mL^−1^. After incubation with PB-NP solutions, absorbance was measured, and the sensor response was evaluated as ΔA = A_0_ − A, where A_0_ is the absorbance before analyte addition, and A is the absorbance after analyte addition. The results were compared to determine whether a significant color change and a decrease in absorbance occurred specifically in the presence of phenylephrine.

The sensor’s reliability was further evaluated through repeatability and stability analyses. For repeatability tests, phenylephrine solutions at 50 µg mL^−1^ and 100 µg mL^−1^ were analyzed under optimized conditions with five consecutive measurements, and the mean absorbance, standard deviation (SD), and relative standard deviation (%RSD) values were calculated. For stability evaluation, PB-NP solutions interacting with 50 µg mL^−1^ phenylephrine were monitored over 42 days (6 weeks), with absorbance measurements recorded at 0, 7, 15, 30, and 42 days, while the solutions were stored at room temperature in the dark between measurements. These analyses enabled the evaluation of both the short-term repeatability and long-term stability of the colorimetric sensor and provided insight into its practical applicability and shelf-life. The measurements were performed in triplicate, and the results are expressed as mean ± standard deviation to account for experimental variability.

### 2.6. Integration of the Colorimetric Sensor into a Paper-Based Platform and Digital Image Analysis

**Scheme 1:**
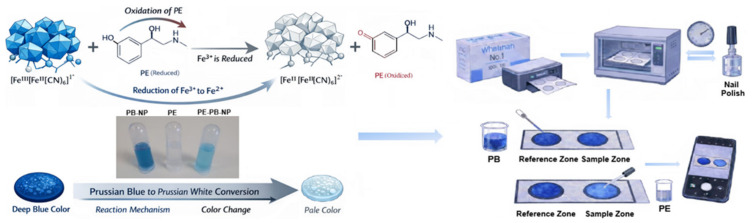
Paper-based colorimetric sensor fabrication and sensing mechanism through redox-induced color change from Prussian blue to Prussian white, and smartphone-based analysis.

The overall preparation of the paper-based sensor, the colorimetric detection process, and the underlying reaction mechanism are illustrated in Scheme 1. The sensors were fabricated using Whatman No. 1 filter paper cut into 25 × 50 mm strips. On each strip, two circular test zones—one for the sample and the other for the reference—were created with an inner diameter of 7.5 mm, leaving at least 5 mm from the edges. To generate hydrophobic barriers, the test patterns were first printed on the paper surface with an inkjet printer, and the printed paper was heated to 120 °C for 5 min to allow the ink to penetrate the porous paper structure and impart hydrophobic properties. However, the resulting barriers were not sufficient to completely prevent liquid spreading. Therefore, the printed circular boundaries were manually coated with nail polish and dried in an oven at 80 °C for 10 min, and this procedure was repeated twice to strengthen the hydrophobic barriers and ensure that the sample solutions remained confined within the defined test zones [18]. Although the hydrophobic barrier was manually formed using nail polish, the procedure provided consistent fluid confinement across different sensors, indicating acceptable reproducibility for practical applications. The synthesized functional nanoparticle suspension was then deposited onto both test zones at a fixed volume of 10 µL, and the paper sensors were allowed to dry at room temperature for 12 h before being used in phenylephrine interaction studies.

The color changes in the paper-based sensors were recorded using the rear camera of a smartphone (Samsung Galaxy S20, 12 MP). All images were captured under controlled ambient lighting conditions at a fixed distance and angle without the use of flash. To ensure consistency and reproducibility, all measurements were performed in the same environment under identical imaging conditions.

The acquired images were analyzed using ImageJ software (version 1.54, National Institutes of Health, USA). The sensing region was selected as the region of interest (ROI), and background areas were excluded from the analysis. The images were converted to 8-bit grayscale to minimize variations associated with RGB channels and lighting conditions. The mean gray intensity values within the ROI were calculated, and background correction was applied to obtain reliable analytical signals. The sensor response was quantified as the change in gray intensity (Δ_gray_) at different phenylephrine concentrations. All measurements were performed in triplicate, and the results are expressed as mean ± standard deviation (SD) [19,20,21].

### 2.7. Real Sample Analysis

An artificial urine matrix was used as a controlled and reproducible medium to evaluate the performance of the developed colorimetric sensor while minimizing potential matrix interferences. The artificial urine solution was prepared according to literature procedures using the components and amounts listed in Table S1 [22,23]. The required chemicals were sequentially added to approximately 80 mL of distilled water and stirred at 350 rpm at room temperature until fully dissolved. The final volume was adjusted to 100 mL, and the pH was set to 6.5. The solution was filtered through a 0.45 µm membrane prior to analysis. The prepared artificial urine solution was stored at 4 °C until use.

## 3. Results and Discussion

### 3.1. Characterization of PB-NPs

#### 3.1.1. Optical Characterization

The interaction between PB-NPs and PE reduces Fe^3+^ ions in the PB structure, leading to the formation of Prussian white, the colorless reduced form of Prussian blue. This transformation results in a significant decrease in the system’s measured absorbance [9]. Since phenylephrine exhibits a maximum absorbance around 260 nm, no characteristic phenylephrine peak was observed within the 400–900 nm wavelength range examined in this study [24]. However, upon interaction between PE and PB-NPs, the intensity of the characteristic PB absorbance peak at 708 nm decreased, and the peak position shifted to 717 nm. This phenomenon corresponds to a redshift in the absorption spectrum and is visually confirmed by the fading of color observed during the formation of Prussian white (Figure S1).

The observed decrease in absorbance intensity together with the red shift from 708 nm to 717 nm suggests an alteration in the electronic structure of PB-NPs associated with the partial reduction in Fe^3+^ centers within the Fe^2+^–CN–Fe^3+^ framework. Phenylephrine contains an electron-donating phenolic hydroxyl group and a secondary amine that increase the electron density of the aromatic ring, facilitating oxidation of PE and concurrent reduction in Fe^3+^ centers in PB under near-neutral pH conditions. The thermodynamic feasibility of this process is supported by the well-established reduction potential of the PB/PW couple (~+0.20 V vs. NHE), which reflects the strong tendency of Fe^3+^ centers in PB to accept electrons [25]. Voltammetric studies have confirmed the electrochemical oxidizability of PE at modified electrode surfaces under mildly acidic conditions [26], consistent with its role as an electron donor in the PB–PW redox transition. Although UV–Vis results alone cannot provide direct electrochemical evidence of PB reduction, the combined observations of absorbance decrease, spectral red shift, visual color fading, and surface interaction evidence from FTIR and XPS analyses collectively support the occurrence of a phenylephrine-induced PB–PW transformation.

#### 3.1.2. Morphological and Structural Characterization

**Figure 1:**
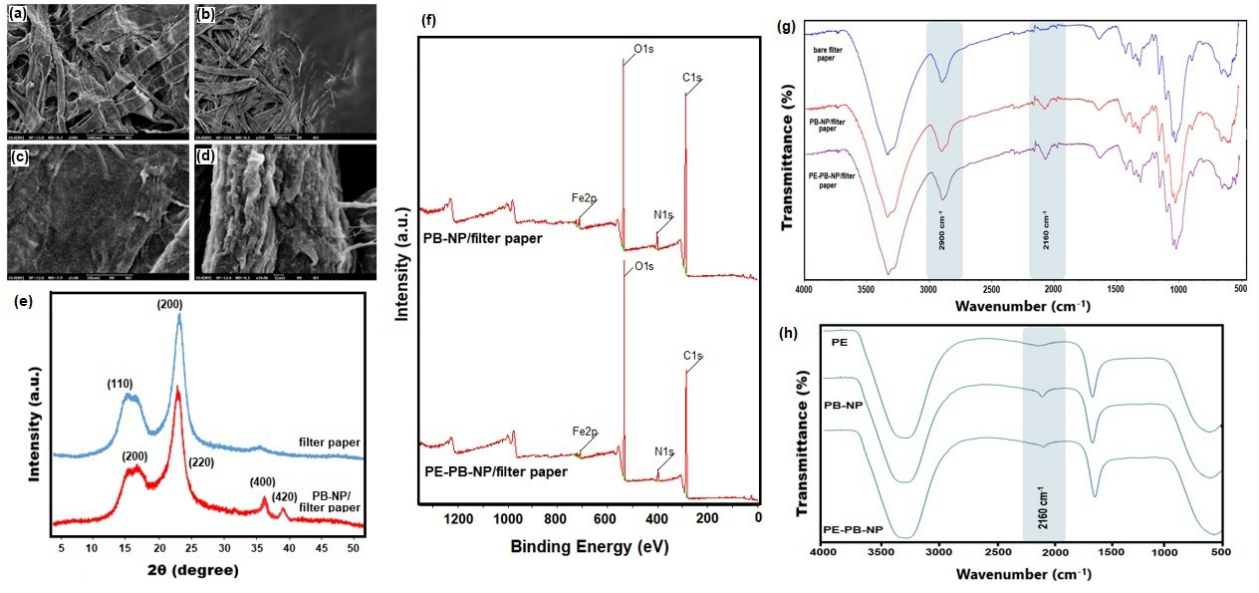
(**a**–**d**) SEM images of unmodified filter paper and PB-NP-modified filter paper surfaces. (**e**) XRD patterns of filter paper and PB-NP/filter paper. (**f**) XPS survey spectra of PB-NP/filter paper and PE–PB-NP/filter paper. (**g**,**h**) FTIR spectra of paper-supported samples and colloidal systems, respectively.

Scanning electron microscopy (SEM) analyses were performed to examine the morphological differences between the unmodified filter paper, the hydrophobic barrier formed using nail polish, and the PB-NP-modified filter paper surface. The SEM images revealed that applying nail polish significantly altered the porous cellulose network of the filter paper. In the unmodified paper, randomly oriented cellulose fibers and interconnected macroporous capillary channels were clearly observed (Figure 1a), whereas the nail polish layer formed a continuous polymeric film that partially blocked these capillaries, thereby creating a hydrophobic barrier and limiting liquid penetration (Figure 1b). This behavior is consistent with previous studies reporting that nitrocellulose-based nail polish coatings generate a low-surface-energy hydrophobic phase on paper substrates [18,27]. Furthermore, while the unmodified filter paper exhibited distinct cellulose microfibrils forming a porous fibrous network, the PB-NP-modified surface showed increased surface roughness, nanoparticle clusters, and a denser coverage layer on the cellulose fibers (Figure 1c,d), indicating successful attachment of PB-NPs onto the paper surface and an increase in the effective surface area [19].

The crystalline structure of the PB-NPs on the paper sensor surface was investigated by X-ray diffraction (XRD) analysis. The XRD patterns of the unmodified filter paper and PB-NP-modified filter paper are presented in Figure 1e. The bare filter paper exhibited the typical semi-crystalline structure of cellulose, with a broad shoulder at 2θ ≈ 15.9° and a prominent peak at 2θ ≈ 22.8°, corresponding to the (110) and (200) planes of cellulose, respectively [28]. The broad and asymmetric nature of these peaks indicates the presence of amorphous regions and irregular fiber packing. After PB-NP modification, characteristic diffraction peaks of Prussian blue appeared at approximately 2θ ≈ 17.4° and 24.7°, corresponding to the (200) and (220) planes, which partially overlap with the cellulose reflections. The relative intensity of this region decreased, likely due to surface coverage of cellulose fibers by PB-NPs. In addition, distinct and sharper peaks were observed around 35.3° and 39–40°, which can be assigned to the (400), and (420) planes of the face-centered cubic structure of Prussian blue (Fe_4_[Fe(CN)_6_]_3_·xH_2_O), confirming the successful deposition of PB-NPs on the filter paper surface [29].

#### 3.1.3. Chemical and Elemental Characterization

To distinguish the chemical interactions occurring in PB-NP and PE-containing PB-NP systems from possible effects originating from the paper matrix, and to evaluate whether the nanoparticles preserved their structural integrity during transfer from the colloidal phase to the paper-based sensor surface, FTIR spectra of both paper-supported and colloidal samples were comparatively analyzed (Figure 1g,h). The FTIR spectrum of the unmodified filter paper exhibited a broad band at 3300–3400 cm^−1^, corresponding to the stretching vibrations of cellulose –OH groups (Figure 1g). A weak band around 2900 cm^−1^ was attributed to C–H stretching vibrations, while the strong band observed at 1050–1030 cm^−1^ corresponds to the C–O–C and C–O stretching vibrations of cellulose. For the PB-NP-modified filter paper, the characteristic cellulose bands were largely preserved. However, a distinct band appearing at 2100–2000 cm^−1^ was observed, corresponding to the C≡N stretching vibration (Fe–CN–Fe) characteristic of Prussian blue nanoparticles. The absence of significant shifts in the main cellulose bands suggests that PB-NPs interact with the paper matrix mainly through physical adsorption and hydrogen bonding rather than covalent bonding [25]. In the PE-containing PB-NP-modified paper, a decrease in the intensity of the –OH band (~3330 cm^−1^) and C–O bands (1200–1000 cm^−1^) was observed compared with the PB-NP-modified sample, indicating interactions between PE molecules and the PB-NP surface via hydrogen bonding. In addition, spectral variations around ~1640 cm^−1^ can be attributed to interactions between the aromatic and heteroatom-containing functional groups of PE and the PB-NPs. Changes in the intensity and shape of the 2100–2000 cm^−1^ band further support the interaction between PE molecules and the nanoparticle surface while indicating that the functional structure of PB-NPs remains preserved.

The FTIR spectra of the colloidal samples (Figure 1h) provide additional insight into these interactions. In the spectrum of PE, the broad band at 3300–3400 cm^−1^ corresponds to phenolic –OH stretching vibrations, broadened due to hydrogen bonding in the aqueous medium. Bands around 1600 cm^−1^ are attributed to aromatic C=C stretching vibrations and N–H bending vibrations of secondary amine groups. For the PB-NP colloid, the broad band at 3330–3340 cm^−1^ corresponds to O–H stretching vibrations from water molecules and surface hydroxyl groups. The band observed at 1600–1650 cm^−1^ is associated with asymmetric stretching vibrations of carboxylate (COO^−^) groups originating from citrate used as a stabilizer during synthesis. The characteristic bands observed in the 2160–2080 cm^−1^ region correspond to the C≡N stretching vibrations of the PB structure [30]. In the PE–PB-NP colloidal spectrum, the characteristic C≡N stretching band (~2165 cm^−1^) remains present but shows a decrease in intensity compared with the pure PB-NP spectrum, indicating that the Prussian blue crystal structure remains intact while PE molecules interact with the nanoparticle surface. Additional changes observed in the 1600–1500 cm^−1^ region and the O–H band (~3340 cm^−1^) suggest the formation of hydrogen bonding and weak electrostatic interactions between PE molecules and the PB-NP surface. The FTIR results indicate that the PE–PB-NP interaction is preserved in the paper-based system, while the retention of characteristic PB-NP bands, particularly the C≡N stretching vibration, and the absence of significant band shifts suggest that the interaction occurs mainly through physical adsorption and hydrogen bonding, confirming the structural stability of PB-NPs for paper-based sensor applications.

The surface chemical composition of the PB-NP-modified filter paper and the phenylephrine (PE)-interacted PB-NP/filter paper samples was investigated using X-ray photoelectron spectroscopy (XPS) (Figure 1f and Table S2). The relatively high carbon and oxygen percentages are attributed to the cellulose-based paper substrate. In the PB-NP/filter paper sample, the atomic percentages of Fe and N were determined as 2.98% and 4.30%, respectively. The Fe2p peak observed at approximately 707.86 eV confirms that the characteristic Fe–C≡N coordination structure of Prussian blue is preserved. The N1s peak at 397.0 eV corresponds to the metal-coordinated cyanide group, further verifying the successful immobilization of PB-NPs on the paper surface [31].

For the PE-interacted PB-NP/filter paper sample, the Fe atomic percentage decreased slightly to 2.55%, and the Fe2p peak was detected at approximately 707.84 eV. A small shift of 0.02 eV in the Fe binding energy was observed. Additionally, a slight increase of 0.03% in the N atomic percentage and a 0.01 eV shift in the N binding energy were detected. Although the observed binding energy shifts were minor, the changes in relative elemental composition and the preservation of Fe–CN-related signals suggest surface interaction without disruption of the PB framework. Moreover, after the addition of phenylephrine, a decrease in the C1s percentage and a notable increase in the O1s percentage were observed. The small variation in the N1s percentage is attributed to the normalization of the relative surface composition.

Overall, the XPS results indicate that phenylephrine does not disrupt the crystal structure of PB-NPs but does interact with the nanoparticle surface, slightly modifying its surface electronic environment. These findings provide supporting evidence for the surface interaction between PE and PB-NPs.

The aqueous suspensions of PB-NPs and PE-PB-NPs were characterized by dynamic light scattering (DLS) (Figure S2). The hydrodynamic diameter of PB-NPs was determined to be 189.0 nm (PdI = 0.246), whereas the PE–PB-NP sample showed a slightly smaller size of 171.3 nm (PdI = 0.266). The presence of a single dominant peak in both samples and PdI values below 0.3 indicate that the systems are relatively monodisperse and stable. The zeta potential of PB-NPs was measured as −34.8 mV, while after interaction with PE the negative surface charge was preserved (−35.7 mV), although changes in the mobility distribution were observed. This suggests that PE adsorption does not occur through strong electrostatic attraction but rather through hydrogen bonding, π–π interactions, or surface complexation, leading to a rearrangement of the surface charge density and the electrical double layer [11]. Since the electrical double layer consists of a Stern layer and a diffuse layer surrounding the particle surface, and the hydrodynamic diameter measured by DLS includes both the hydration shell and this double layer, the slight decrease in particle size can be attributed to the compaction of the electrical double layer following surface interaction.

### 3.2. Optimization of the Colorimetric Sensor

The effect of PB-NP volume on the analytical response was investigated by recording the UV–Vis spectra of different volumes of the synthesized PB-NP colloid. The increasing PB-NP volume led to a gradual increase in absorbance intensity, while maintaining the characteristic absorption band of PB around 700 nm (Figure S3). However, excessively high nanoparticle volumes produced very strong absorbance signals, potentially reducing the sensitivity of the colorimetric response to the analyte. Therefore, an intermediate PB-NP volume of 500 µL was selected as the optimal condition, providing a sufficiently intense and stable analytical signal while remaining within the measurable absorbance range for subsequent experiments.

Investigating the time-dependent variation in the sensor signal is essential for determining the optimum incubation time. To evaluate this effect, a mixture containing a constant amount of PB-NPs (500 µL) and 50 µg mL^−1^ PE in a total volume of 3 mL was incubated for 0–30 min, and the absorbance was recorded at each time point (Figure S4). A rapid decrease in absorbance was observed within the first few minutes after PE addition, indicating the fast formation of Prussian white due to the PB-NP–PE interaction. The signal stabilized after approximately 5 min, suggesting that the system had reached equilibrium. Therefore, 5 min was selected as the optimum incubation time for subsequent experiments.

**Figure 2:**
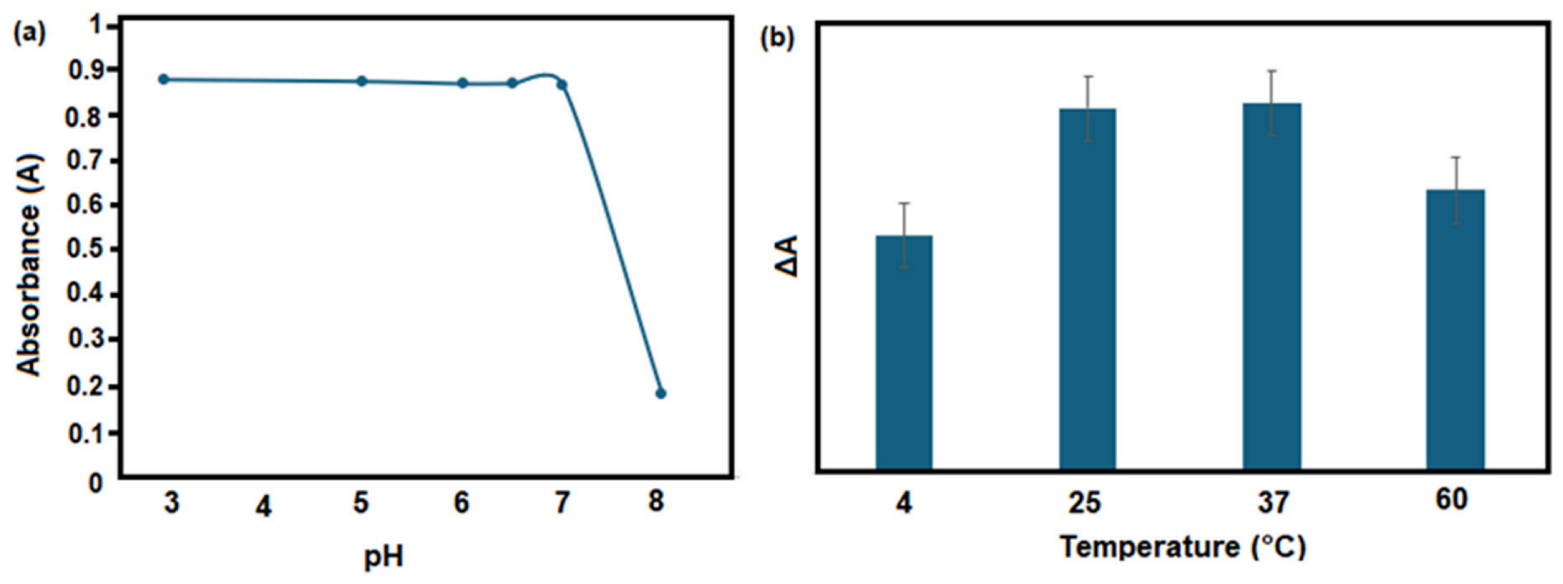
(**a**) Effect of pH on the absorbance response of the PB-NP-based sensor. (**b**) Effect of temperature (°C) on the sensor response (ΔA) toward phenylephrine.

PB-NPs are sensitive to the pH of the surrounding medium due to their metallic structure, which may affect both their structural stability and optical response [9]. To investigate the pH-dependent optical response of the PB-NP–phenylephrine (PE) interaction, buffer solutions with different pH values (pH 3–8) were prepared. In each solution, a constant volume of PB-NPs and a fixed concentration of PE were added, and absorbance was measured at the optimal incubation time. As shown in Figure 2a, only minor changes in absorbance were observed within the pH 3–6.5 range, whereas a significant decrease occurred at pH > 7, which can be attributed to the reduced stability of Fe^3+^ ions and structural degradation of PB-NPs in alkaline conditions [10].

Considering nanoparticle stability, sufficient sensor response, and compatibility with biological matrices such as urine, pH 6.5 was selected as the optimum working pH. The selection of pH 6.5 is also consistent with the redox behavior and structural stability of PB-NPs. Under strongly acidic conditions, protonation may influence the interaction between PE and the PB surface, whereas alkaline conditions may promote hydrolysis of Fe^3+^ species and destabilize the PB framework, resulting in reduced optical stability. Near-neutral pH conditions favor both the preservation of the mixed-valence Fe^2+^/Fe^3+^ structure of PB and the electron-transfer interaction between PE and PB-NPs. In addition, pH 6.5 is within the physiologically relevant range for urine analysis, supporting the practical applicability of the developed sensing platform.

Temperature is another important parameter affecting biosensor responses. The catalytic activity and stability of PB-NP-based sensors may vary with temperature, and excessively high temperatures can lead to chemical instability. Therefore, the interaction between the analyte and PB-NPs was evaluated at different temperatures to determine the optimum condition. For this purpose, the sensor was incubated with phenylephrine solutions at a fixed concentration at 4, 25, 37, and 60 °C for 5 min (optimal incubation time), and the corresponding responses were recorded. The change in absorbance (ΔA) resulting from the interaction between phenylephrine (PE) and PB-NPs was used as the indicator of sensor performance. As shown in Figure 2b, the ΔA values reached their maximum at 25 °C and 37 °C, indicating the most efficient interaction between the sensor and PE at these temperatures. At 60 °C, the ΔA value was slightly lower than that at 25 °C, suggesting that the sensor still retained its interaction capability at elevated temperature. In contrast, at 4 °C, the ΔA value was approximately 0.052, which can be attributed to slower reaction kinetics at low temperatures. Although high interaction efficiency was also observed at 37 °C, 25 °C was selected as the optimum operating temperature due to its practicality for field applications, and subsequent experiments were conducted at this temperature.

### 3.3. Analytical Performance of the PB-NP-Based Colorimetric Sensor

**Figure 3:**
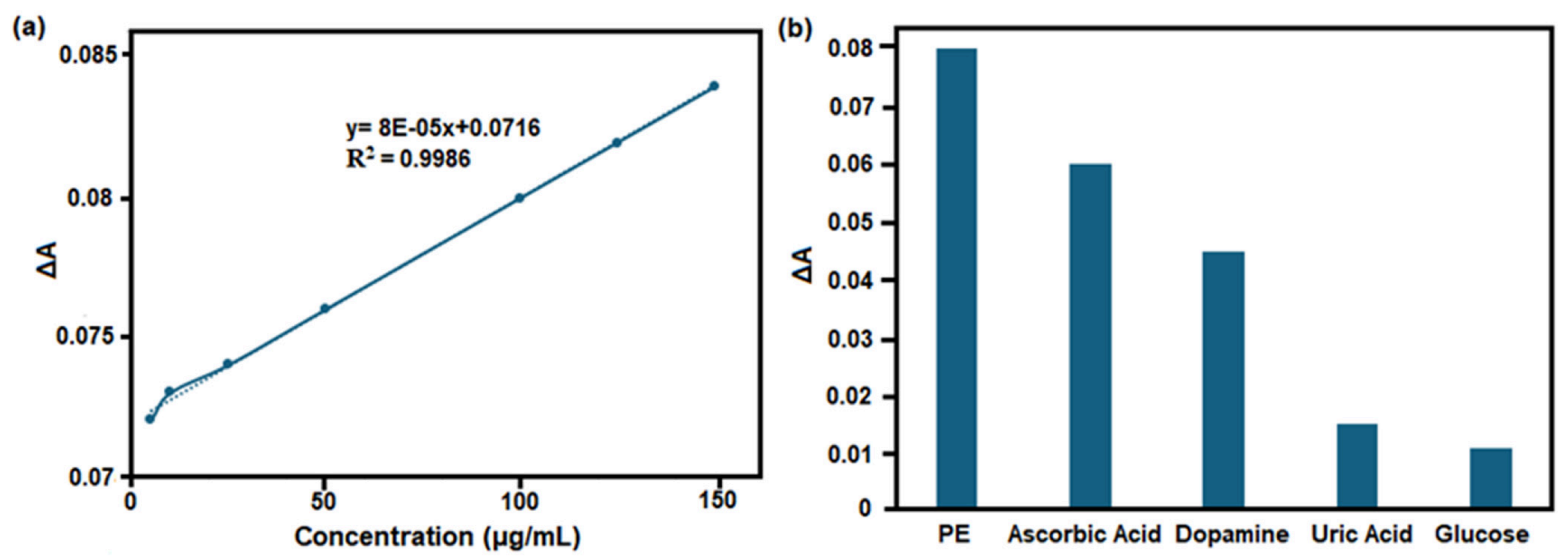
(**a**) Calibration curve of the PB-NP-based colorimetric sensor for phenylephrine (PE) detection. (**b**) Selectivity of the sensor toward PE compared with potential interfering compounds.

Under optimum experimental conditions, the analytical performance of the PB-NP-based colorimetric sensor for the determination of phenylephrine (PE) was evaluated. For this purpose, PB-NP solutions containing 0.5–150 µg mL^−1^ PE were prepared, and their optical responses were measured using a UV–vis spectrophotometer. Although the calibration experiments were conducted over the concentration range of 0.5–150 µg mL^−1^, a linear response was observed only between 5 and 150 µg mL^−1^ (Figure 3a), while deviations from linearity occurred at lower concentrations. The deviations from linearity below 5 µg mL^−1^ may be attributed to the relatively small absorbance changes generated at low phenylephrine concentrations, where the analytical signal approaches the inherent variability of the optical measurement system. Consequently, the signal-to-noise ratio decreases, reducing linearity in the lower-concentration region. The regression equation was calculated as y = 8 × 10^−5^x + 0.0716, with a determination coefficient of R^2^ = 0.9986. This linear behavior demonstrates that the proposed sensor is suitable for the quantitative determination of PE over a relatively wide concentration range. The sensitivity of the sensor was evaluated from the slope of the calibration curve, and the limit of detection (LOD) was calculated as the ratio of three times the standard deviation of the blank (3σ) to the slope of the calibration curve, yielding a value of 1.56 µg mL^−1^.

A systematic comparison with previously reported phenylephrine sensors and spectrophotometric methods is provided in Table S3. An indirect spectrophotometric method has been reported based on the oxidation of phenylephrine using potassium permanganate, followed by the bleaching of toluidine blue dye in acidic medium, with absorbance measured at 606 nm. The method exhibited a linear range of 1.0–10.0 µg mL^−1^ and an LOD of 1.11 µg mL^−1^ when applied to pharmaceutical formulations [32]. Similarly, a UV–Vis method was reported based on azo-coupling with diazotized sulfacetamide sodium, producing a yellow azo dye measured at 425 nm, with a linear range of 2–24 µg mL^−1^, R^2^ = 0.9929, and LOD = 0.278 µg mL^−1^ [33]. In another study, PE was determined using N,N-dimethyl-p-phenylenediamine dihydrochloride (NNDPH) via an oxidative coupling reaction in basic medium with ferric chloride, producing a green-blue dye [34]. The method showed a linear range of 4–22 µg mL^−1^ with a reported LOD of 4 ppm. Likewise, a colorimetric method was developed based on diazotization and azo coupling with clonazepam, producing an orange azo dye measured at 466 nm, with a linear range of 0.5–9 µg mL^−1^, LOD = 0.006 µg mL^−1^, and LOQ = 0.02 µg mL^−1^ [35]. However, the method requires multiple reagents, controlled addition steps, and longer reaction times. In comparison, the PB-NP-based colorimetric sensor developed in this study provides a strong linear response within 5–150 µg mL^−1^ (R^2^ = 0.9986), offering a wider working range and a simpler detection mechanism based on direct color change (Figure 3a). The nanoparticle-assisted signal generation enables sensitive detection even at low to moderate PE concentrations. Therefore, the proposed method represents a practical and competitive alternative for PE determination.

### 3.4. Selectivity of the Sensor

The selectivity of the developed PB-NP-based colorimetric sensor was evaluated by comparing its response to PE with structurally related or biologically relevant compounds, including dopamine, glucose, uric acid, and ascorbic acid. Each compound was tested at a constant concentration of 100 µg mL^−1^, and the decrease in absorbance relative to the pure PB-NP solution was recorded. All potential interfering compounds were tested at the same concentration to enable direct comparison of their effects on the sensor response under identical conditions. The sensor response was expressed as $∆A=A_{0}-A$, where A_0_ is the absorbance of the PB-NP solution before analyte addition and A is the absorbance after interaction. The calculated ΔA values for all compounds are presented in Figure 3b.

Among the tested compounds, phenylephrine exhibited the highest ΔA value, indicating a stronger interaction with PB-NPs. The significant decrease in absorbance results from the formation of Prussian white through the interaction between PE and PB-NPs. Although ascorbic acid, dopamine, and uric acid, which are redox-active molecules, also produced measurable responses due to possible electron transfer with PB-NPs, their signals were considerably lower than that of PE. In contrast, glucose showed minimal signal change, likely due to its relatively neutral character and weak redox activity, leading to negligible interaction with the PB-NP surface.

These results demonstrate that the proposed sensor exhibits high selectivity for phenylephrine, with limited potential interference from other biological molecules. Consequently, the PB-NP-based colorimetric sensor can be considered a reliable analytical platform for the detection of phenylephrine in complex biological matrices, such as urine.

The sensor’s preferential response to PE over the other tested compounds can be attributed to a combination of redox activity and molecular interactions at the PB-NP surface. Although dopamine possesses a catechol moiety with stronger intrinsic reducing capacity than PE, it produced a lower ΔA response, indicating that reducing capacity alone does not determine selectivity. Under the working pH of 6.5, the protonated secondary amine of PE (pKa ~9.0) may contribute to surface affinity through hydrogen bonding and partial dipole interactions with the negatively charged PB-NP surface (zeta potential: −34.8 mV), rather than through strong electrostatic adsorption, consistent with the negligible change in zeta potential observed upon PE interaction (−34.8 to −35.7 mV).

In contrast, ascorbic acid and uric acid, which lack aromatic amine groups, may exhibit weaker surface interaction despite their reducing character. Glucose, having neither a phenolic –OH nor redox-active aromatic groups, produced negligible response. These findings suggest that the observed selectivity is influenced not only by reducing ability, but also by surface interaction characteristics such as electrostatic attraction, hydrogen bonding, and molecular orientation at the PB-NP surface.

### 3.5. Repeatability and Stability of the Sensor

To evaluate the reliability of the developed colorimetric sensor, its repeatability and stability were investigated. For the repeatability test, five consecutive measurements were performed using phenylephrine (PE) solutions at 50 µg mL^−1^ and 100 µg mL^−1^, and the standard deviation (SD) and coefficient of variation (CV) were calculated (Table S4).

For 50 µg mL^−1^, the mean absorbance change (ΔA) was 0.074, with an SD of 0.00114 and a CV of 1.55%. Similarly, for 100 µg mL^−1^, the mean absorbance value was 0.0804, with an SD of 0.00114 and a CV of 1.42%. The CV values being below 2% indicate high precision and good repeatability of the sensor at both concentrations. The slight decrease in CV with increasing concentration suggests improved signal-to-noise ratio and measurement stability at higher analyte levels.

The stability of the sensor was evaluated by monitoring the absorbance of a 50 µg mL^−1^ PE–PB-NP solution over 42 days (6 weeks) (Table S5). During the stability study, the PB-NP solutions were stored in closed containers at room temperature (25 °C) in the dark to minimize light-induced degradation and environmental effects. No additional humidity control was applied during storage. After 42 days, approximately 78.9% of the initial sensor response was retained. Although absorbance decreased gradually over time, the sensor retained approximately 78.9% of its initial analytical response after 42 days, indicating acceptable short-term storage stability for practical colorimetric applications. The observed decrease in the signal may be attributed to partial aggregation or surface oxidation of PB-NPs during storage; however, the system largely maintained its optical stability.

### 3.6. Paper-Based Sensor Application and Digital Image Analysis

To quantitatively evaluate the color change generated by the PB-NP-based paper sensor in the presence of phenylephrine (PE), region-of-interest (ROI)-based digital image analysis was performed using ImageJ, a widely used open-source tool for colorimetric sensor analysis [36]. In image-based colorimetric analysis, converting color variations into numerical values is essential. Since direct use of RGB channels can be sensitive to lighting conditions and hardware differences, the images were converted to 8-bit grayscale, allowing the color intensity to be expressed as a single parameter, the mean gray value, which improves reproducibility and simplifies background correction and quantitative analysis [37]. After importing the images into ImageJ, the sensing region was selected as the ROI, while the surrounding areas were treated as background and excluded from the analysis. The mean gray intensity values of the ROI were calculated, and background values were subtracted to obtain corrected ImageJ signals. These quantitative intensity values were then correlated with PE concentration to construct calibration curves, following commonly used digital image colorimetry (DIC) approaches [38].

**Figure 4:**
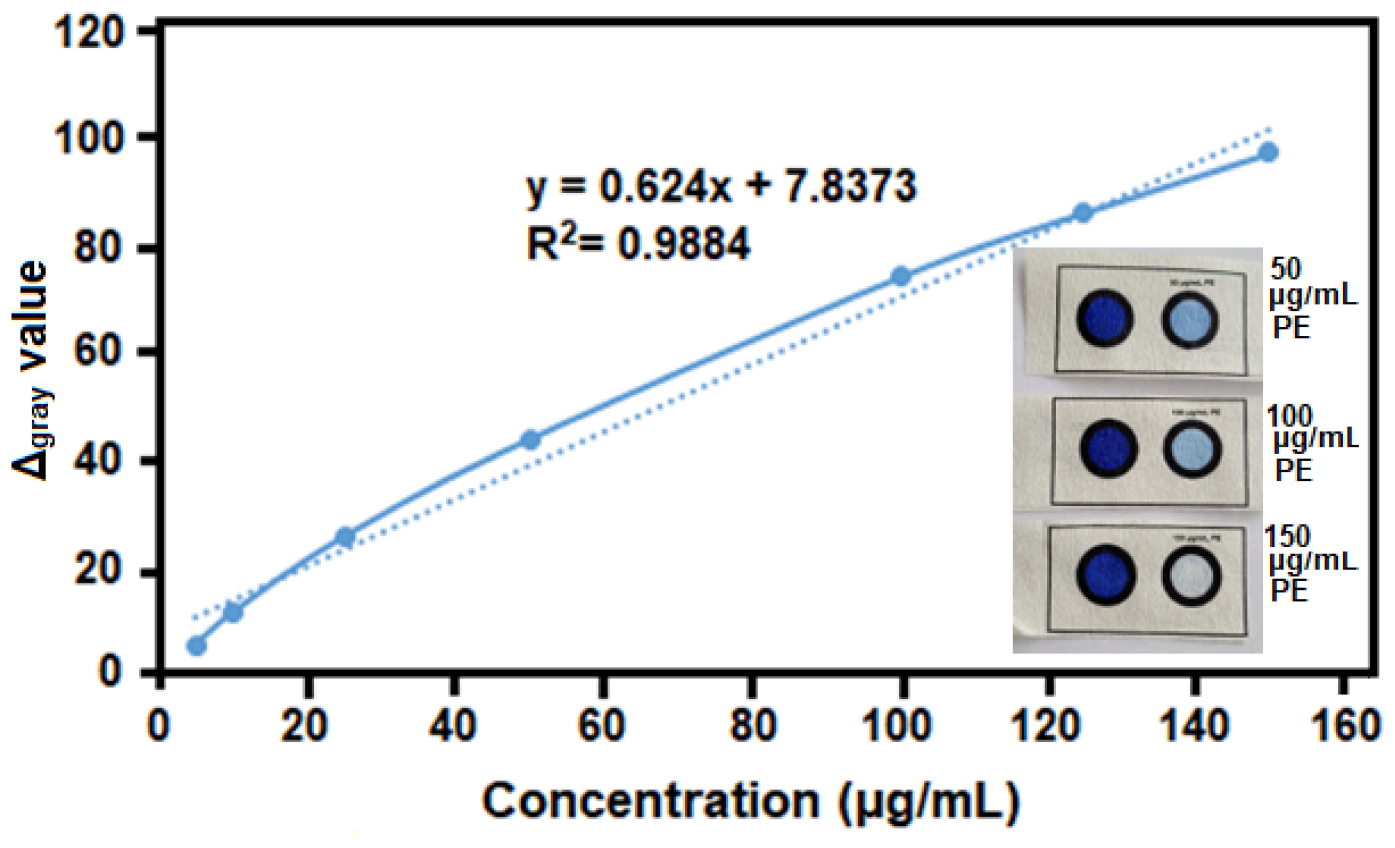
Calibration curve obtained from image-based analysis. Inset: representative color changes at different concentrations.

For this purpose, PE solutions in the range of 5–150 µg mL^−1^ were dropped onto the paper sensor, and the color change after 5 min of incubation was recorded under natural light using a smartphone camera. As the concentration increased, the blue color of Prussian blue gradually faded as it converted to Prussian white, resulting in a systematic decrease in the gray intensity values calculated by ImageJ, while the reference region remained unchanged. The corresponding ImageJ calibration curve is shown in Figure 4, indicating a linear relationship over 5–150 µg mL^−1^ with a determination coefficient (R^2^) of 0.9884 and a calculated LOD of 5.37 µg mL^−1^.

Although the LOD obtained from the image-based method is higher than that obtained by UV–visible spectrophotometry, likely due to factors such as illumination variability, camera resolution, and the heterogeneity of the paper surface, the ImageJ–ROI approach exhibits a consistent linear trend comparable to the spectrophotometric results [38]. This confirms that the developed PB-NP-based colorimetric sensor produces a reliable visual signal suitable for practical detection.

### 3.7. Sample Analysis

To evaluate the applicability of the developed PB-NP-based paper sensor to real sample matrices, phenylephrine was determined in artificial urine. The artificial urine sample was initially analyzed directly and subsequently spiked with PE standards at concentrations of 50 and 100 µg mL^−1^ for quantitative evaluation using the standard-addition approach (Table S6). Measurements were carried out in triplicate for each concentration, and ΔGray values were calculated using ROI-based image analysis with ImageJ software. The data were obtained using background-corrected gray intensity values.

In the standard-addition experiments performed at 50 and 100 µg mL^−1^, the recovery values were calculated as 97.5% and 95.87%, respectively. The fact that the recovery values fall within the acceptable range of 95–105% indicates that the method exhibits satisfactory accuracy. Moreover, the relative standard deviation (%RSD) values of 2.13% and 1.15% demonstrate good repeatability and adequate precision of the developed paper-based colorimetric sensor. These results confirm that the proposed method is applicable for reliable and quantitative determination of PE in artificial urine matrices.

## 4. Conclusions

In this study, a Prussian blue nanoparticle (PB-NP)-based colorimetric sensor was developed for the determination of phenylephrine, and its analytical performance was evaluated. The colorimetric sensor exhibited a strong linear response over the concentration range of 5–150 µg mL^−1^, with a high correlation coefficient (R^2^ = 0.9986), enabling sensitive detection at low–moderate concentrations. Selectivity studies demonstrated that the sensor shows high specificity toward phenylephrine, while common interferents such as ascorbic acid, dopamine, uric acid, and glucose did not significantly affect the sensor response. Future studies involving structurally related compounds, such as ephedrine, pseudoephedrine, and adrenaline, may provide further insight into the mechanism of selectivity and broaden the applicability of the proposed sensing platform. The sensor also exhibited good repeatability with variation coefficients below 2%, and retained approximately 78.9% of its initial signal after 42 days, indicating satisfactory stability. By integrating the colorimetric system into a paper-based sensing platform and applying ImageJ-based digital image analysis, quantitative evaluation was successfully achieved. In artificial urine samples, the standard-addition method yielded recovery values between 95% and 97.5%.

Compared with conventional colorimetric methods that often require multiple reagents and multi-step procedures, the proposed sensor provides a wider linear working range and a simple color change-based detection mechanism. Although advanced nanosensor systems, such as electrochemical and fluorescence-based sensors, may provide lower detection limits for phenylephrine, they often require complex instrumentation, longer analysis times, and sophisticated fabrication procedures. In contrast, the developed platform offers a simple, rapid, cost-effective alternative with sufficient sensitivity and potential for practical applications, particularly for on-site and point-of-care analysis. Furthermore, to the best of our knowledge, this study represents the first application of a PB-NP-based paper colorimetric sensor exploiting the Prussian blue–Prussian white transition for phenylephrine determination.

## Data Availability

The original contributions presented in this study are included in the article/Supplementary Materials. Further inquiries can be directed to the corresponding author.

## Supplementary Materials

The following supporting information can be downloaded at: https://www.mdpi.com/article/10.3390/bios16060339/s1. Table S1: Components and amounts used for the preparation of the artificial urine solution; Figure S1: UV–Vis absorption spectra of PB-NP, PE, and PE–PB-NP systems with the corresponding photographic image of the samples; Table S2: XPS binding energies and atomic percentages of elements; Figure S2: Hydrodynamic size distributions of (A) PB-NP and (B) PE-PB-NPs; Figure S3: UV–Vis absorption spectra of PB-NPs at different volumes and Inset: Absorbance vs. volume plot; Figure S4: Optimization of incubation time; Table S3: Comparison of previously reported colorimetric methods for phenylephrine determination; Table S4: Repeatability results of the colorimetric sensor (*n* = 5); Table S5: Stability data of the sensor over time; Table S6: Recovery (%) and relative standard deviation (%RSD) values obtained by the standard addition method in artificial urine samples (*n* = 3).

## Abbreviations

The following abbreviations are used in this manuscript:

PE	Phenylephrine
PB	Prussian blue
PB-NPs	Prussian blue nanoparticles
