# Supplementary material for: Selective Colorimetric Determination of Phenylephrine Using a Prussian Blue Nanoparticle-Modified Paper-Based Sensor
Source: Biosensors (Basel). 2026 Jun 16;16(6):339. doi: 10.3390/bios16060339 (PMC13297358; doi:10.3390/bios16060339)
Supplement: Supplementary file 1 [file biosensors-16-00339-s001.zip › biosensors-4325498-supplementary.pdf]

Table S1. Components and amounts used for the preparation of the artificial urine solution [22,23].

**Compound**	**Weight (g/100 mL)**
Na_2SO_4	0,1700
Uric acid ($C_5H_4N_4O_3$)	0,0250
Sodium citrate ($Na_3C_6H_5O_7 \cdot 2H_2O$)	0,0720
Creatinine ($C_4H_7N_3O$)	0,0881
Urea (CH_4N_2O)	1,5000
KCl	0,2308
NaCl	0,1756
$CaCl_2$	0,0185
NH_4Cl	0,1266
$NaH_2PO_4 \cdot 2H_2O$	0,2912
$Na_2HPO_4 \cdot 2H_2O$	0,0831

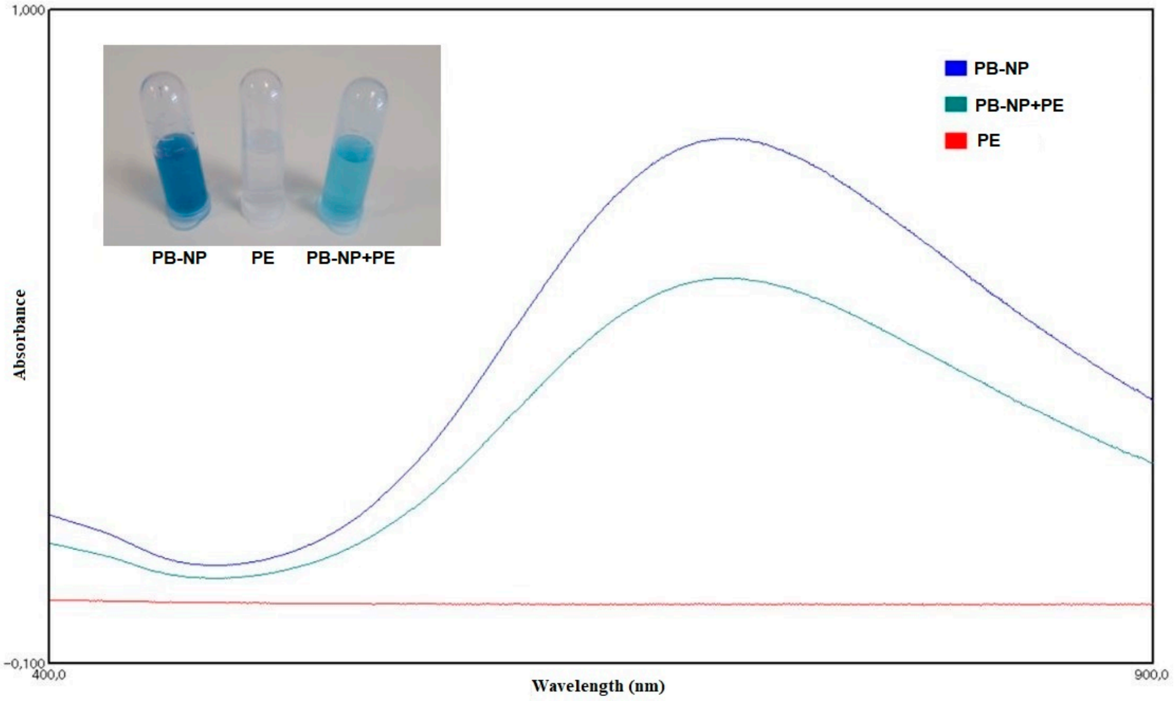

Figure S1. UV–Vis absorption spectra of PB-NP, PE, and PE–PB-NP systems with the corresponding photographic image of the samples.

Table S2. XPS binding energies and atomic percentages of elements.

	PB-NP-modified filter paper		PE-PB-NP-modified filter paper	
**Element**	**Binding Energy (eV)**	**Atomic Percentage (%)**	**Binding Energy (eV)**	**Atomic Percentage (%)**
C1s	284.21	66.35	285.89	55.00
O1s	532.44	26.37	532.47	37.40
N1s	397.00	4.30	397.01	4.33
Fe2p	707.86	2.98	707.84	2.55

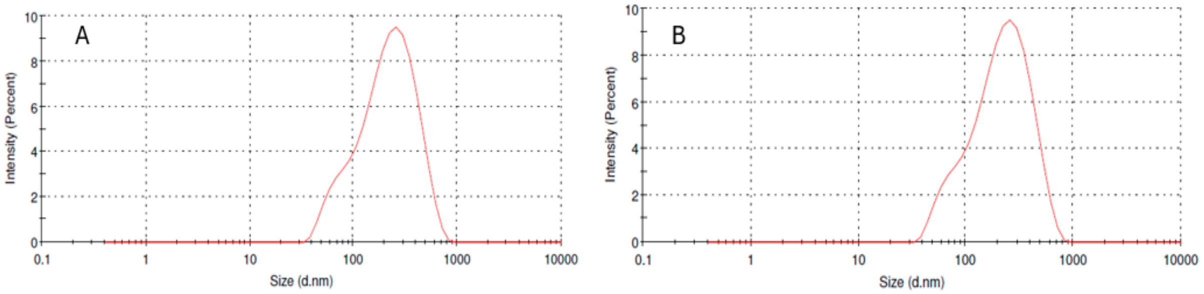

Figure S2. Hydrodynamic size distributions of (A) PB-NP and (B) PE-PB-NPs.

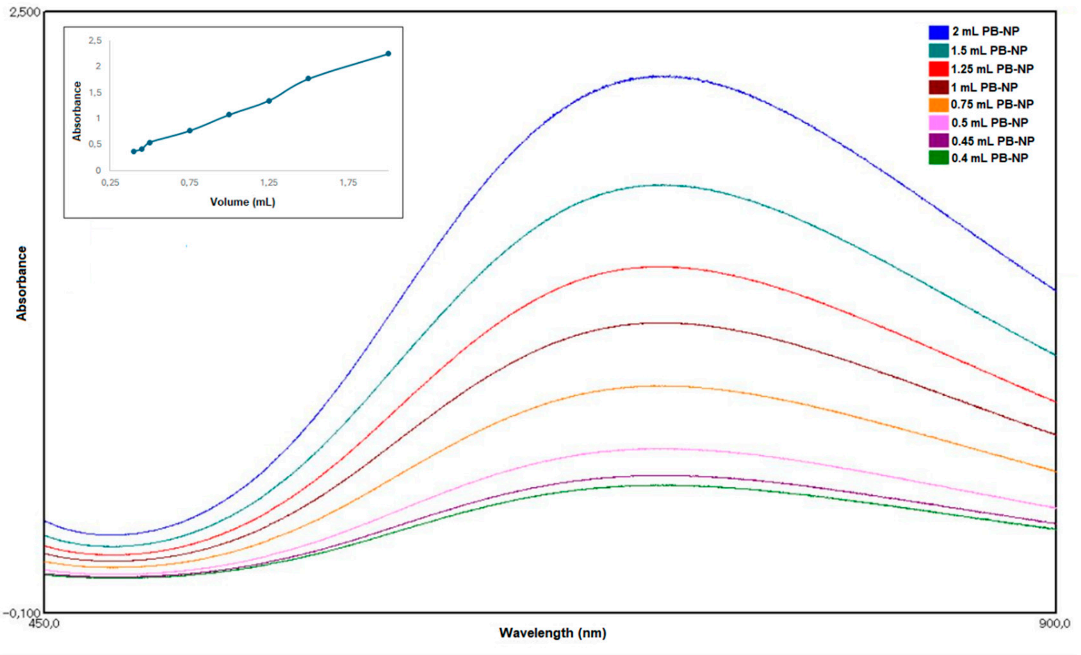

Figure S3. UV–Vis absorption spectra of PB-NPs at different volumes and *Inset:* Absorbance vs. volume plot.

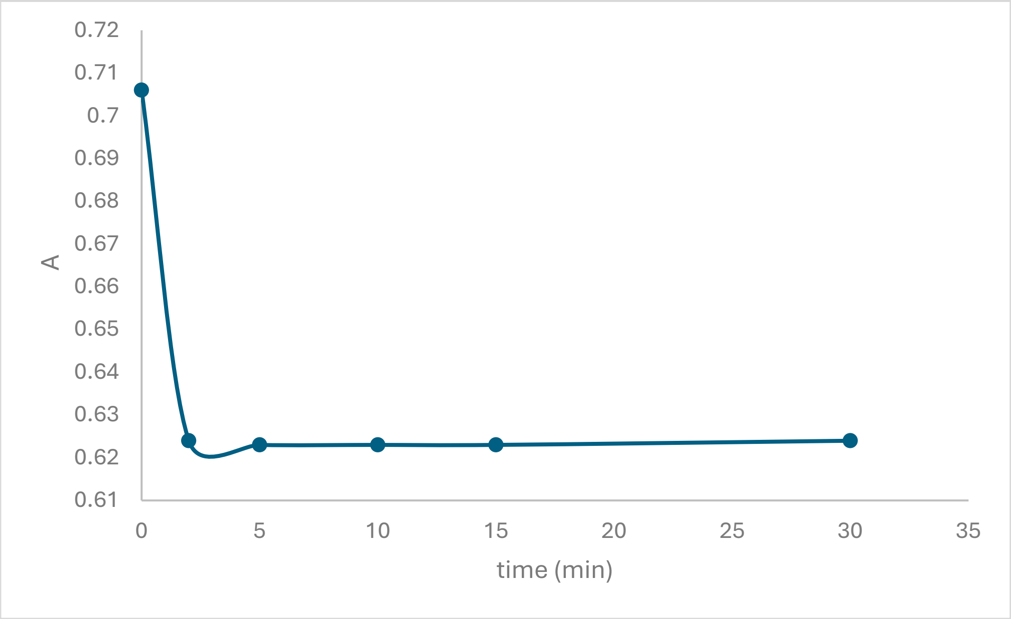

Figure S4. Optimization of incubation time.

Table S3. Comparison of previously reported colorimetric methods for phenylephrine determination.

**Method**	**Modification**	**Limit of Detection**	**Linear Range**	**Reference**
Colorimetric sensor	Oxidation with $KMnO_4$ and toluidine blue dye	1.11 µg/mL	1.0–10.0 µg/mL	[32]
Colorimetric sensor	Azo dye formation	0.278 µg/mL	2–24 µg/mL	[33]
Colorimetric sensor	N,N-dimethyl-p-phenylenediamine with ferric chloride	4 µg/mL	4–22 µg/mL	[34]
Colorimetric sensor	Azo dye formation	0.006 µg/mL	0.5–9 µg/mL	[35]
Colorimetric sensor	Using Prussian blue nanoparticles	1.56 µg/mL	5–150 µg/mL	This study

Table S4. Repeatability results of the colorimetric sensor (n = 5).

50 μg/mL PE		100 μg/mL PE	
**Measurement No**	**ΔA**	**Measurement No**	**ΔA**
1	0.074	1	0.080
2	0.072	2	0.082
3	0.075	3	0.079
4	0.073	4	0.081
5	0.074	5	0.080
Mean	0.0736	Mean	0.0804
Standard Deviation	0.00114	Standard Deviation	0.00114
Coefficient of Variation (%)	1.55	Coefficient of Variation (%)	1.42

Table S5. Stability data of the sensor over time.

Day	ΔA	Signal Retention (%)
0	0.073	100
7	0.072	98.6
15	0.071	97.2
30	0.069	94.5
42	0.058	78.9

Table S6. Recovery (%) and relative standard deviation (%RSD) values obtained by the standard addition method in artificial urine samples (n = 3).

Added PE (μg/mL)	Measured PE (μg/mL)	Recovery (%)	RSD (%)
50	48.75	97.5	2.13
100	95.87	95.87	1.15
